# Undergraduate medical education and pediatric surgery

**DOI:** 10.4103/0971-9261.69146

**Published:** 2010

**Authors:** P. Madhok

**Affiliations:** Ashwini Nursing Home, 15^th^ Road, Khar, Mumbai – 400 052, India. E-mail: drpmadhok@yahoo.com

Sir,

Please accept my congratulations for having the Journal indexed. It must have been a Herculean task.

I read with interest the Editorial by Prof. B. Mukopadhyay on undergraduate medical education in the July–Sept ’09 issue of JIAPS.[1]

I appreciate my fellow Pediatric Surgeons for their efforts to introduce teaching of Pediatric Surgery at the undergraduate level. It is extraordinary that the Committee could meet the Prime Minister in this connection.

However, I am not sure if teaching the entire Pediatric Surgical curriculum is advisable at the undergraduate level. I have been lecturing regularly to the DCH students and the undergraduates for the past several years and I find the following topics sufficient:

1. Neonatal surgical emergencies.
2. Common surgical problems in children.
3. Tracheo-oesophageal foreign bodies.
4. Hydrocephalus–meningomyelocele.
5. Recurrent urinary infection.
6. Mass in abdomen.

Acute abdomen in children. It is important that the graduate does not miss to study life-threatening lesions, especially if amenable to surgery. Burns and trauma are adequately dealt with by adult surgeons. I feel the overburdened undergraduate should not be further burdened, especially when other super specialists, such as Neuro, Plastic, and Bariatric surgeons want a share in undergraduate teaching. Also if we have to take Pediatric Surgery, particularly Neonatal Surgery further, we have to work closely with the Neonatologist. Asking for a section in Pediatric Surgery in the Pediatric Medicine paper might sound more logical than in the General Surgery paper.

I fully agree that a Pediatric surgeon must be available in every medical college and even at the District Hospitals to deal with the surgical needs of children.
